# Cost-effective design of economic instruments in nutrition policy

**DOI:** 10.1186/1479-5868-4-10

**Published:** 2007-04-04

**Authors:** Jørgen D Jensen, Sinne Smed

**Affiliations:** 1 Institute of Food and Resource Economics, Faculty of Life Sciences, University of Copenhagen, Rolighedsvej 25, DK-1958 Frederiksberg C, Denmark; 2 Danish Institute of Governmental Research (AKF), Nyropsgade 37, DK-1602 København V, Denmark

## Abstract

This paper addresses the potential for using economic regulation, e.g. taxes or subsidies, as instruments to combat the increasing problems of inappropriate diets, leading to health problems such as obesity, diabetes 2, cardiovascular diseases etc. in most countries. Such policy measures may be considered as alternatives or supplements to other regulation instruments, including information campaigns, bans or enhancement of technological solutions to the problems of obesity or related diseases. 7 different food tax and subsidy instruments or combinations of instruments are analysed quantitatively. The analyses demonstrate that the average cost-effectiveness with regard to changing the intake of selected nutritional variables can be improved by 10–30 per cent if taxes/subsidies are targeted against these nutrients, compared with targeting selected food categories. Finally, the paper raises a range of issues, which need to be investigated further, before firm conclusions about the suitability of economic instruments in nutrition policy can be drawn.

## 1. Background

Inappropriate diets call for increasing concern in many industrialised countries because they increase the risk of a range of diseases, including obesity, diabetes 2, cardiovascular diseases, etc. For example, during the last 40–50 years, the occurrence of obesity has increased considerably. Among the reasons could be mentioned a high supply of cheap foods, a change in the composition of the diet in the direction from vegetables towards more saturated fats and sugar, a change towards less physical activity which has not been accompanied by a corresponding reduction in the energy intake, and an increasing consumption of convenience foods and prepared fastfood meals [1-3]. The technological development through the last decades has lowered the costs of acquiring calories and increased the costs of expending these calories^i^. Hence, the relative price between physical activity and calories has changed and consequently reduced the economic incentives to a healthy balance between food intake and physical activity [3]. Inappropriate diets are not only a health problem but also an economic problem, because many of these diseases induce considerable costs for society [4-7].

Several suggestions have been made in order to reduce the fraction of people with food intakes deviating substantially from dietary recommendations and thus to counter the challenge from diet-related health risks. Suggested measures include information campaigns, tighter rules for advertising, promotion of healthier eating at schools, modified food taxes or subsidies, etc. [8]. The idea behind modified food taxes or subsidies is to provide consumers with economic incentives to change their food consumption in a direction towards nutritional recommendations, thus reducing the probability of being exposed to obesity and other health risks. However, in contrast to tobacco and alcohol, which have been subject to special taxation for many years in many countries, the use of differentiated food taxes or subsidies has not been heavily represented on the agenda with respect to nutritional objectives, and empirical experience with regard to differentiated food taxes – and thus empirical evidence about the effects of food taxation on food consumption and health – is practically non-existing. Despite the lack of empirical evidence, the following causality chain could however be presumed: tax change → food prices → food consumption → fraction of people deviating from nutritional recommenation → fraction of people exposed to health risks. The objective of the present paper is to illuminate the quantitative potentials of differently targeted food subsidies or taxes as instruments to improve diets and hence reduce the fraction of people exposed to diet-related health risks. This goal is pursued in order to evaluate the importance of proper targeting, if such instruments should be efficient policy measures in the improvement of dietary behaviour in industrialised countries. Denmark is used as an illustrative case.

## 2. Extent of inappropriate dietary behaviour

Increasing prevalence of overweight and obesity in most countries suggest that the actual diets are not matching the prevailing nutritional recommendations [9-11]. As an example, table 1 shows the intake of selected nutrients for Danish adults and children. Fats' share of total energy intake is above the recommended level for both adults and children, whereas children's intake of sugar exceeds the recommended level. Different studies suggest that food consumption patterns as well as the problems of obesity and bad diets vary according to age, level of education and region [12-17]. Hence, the frequency of obesity is relatively high among people in rural areas and people with lower levels of education. Elderly people tend to consume too much saturated fat, while younger people tend to consume too much sugar.

**Table 1:** Danish consumers' intake of nutrients per day, 2000/01

	Children 4–14 years	Adults 15–75 years	Recommended
Energy (MJ)	8,5	9,2	
Fat (E%)	34	34	30
Fat (g/day)	75	79	
Carbohydrate (E%)	53	48	
Sugar (E%)	14,0	9,3	10
Sugar (g/day)	71	52	
Fibres (g/10MJ)	19	22	
Protein (E%)	13	13	
Fruits (g/day)	216	239	
Vegetables (g/day)	117	151	
Potatoes (g/day)	78	110	

Source: [18]

In addition to the problems faced by individuals in terms of bad health, lack of social acceptance and a number of inconveniences, diet-related health problems also induce externality costs to society in terms of public financed costs to health care and reduced productivity. For example, it has been estimated that 5–8 per cent of the total health care budget is used for overweight-related diseases in many industrialised countries [2,15,19,20]. In the prospect of future increases in the occurrence of such health problems, an increase in these costs may also be foreseen.

## 3. Economic policy instruments in nutritional policy

Whereas arguments for nutrition policy intervention can be raised from many perspectives, the existence of externality costs can be considered as the main argument for public intervention from a strictly economic perspective. The theoretical foundation for using economic incentives to regulate diet habits is the assumption that demand curves are downward sloping. Econometric studies for several countries suggest that prices do have an impact on the composition of food consumption, e.g. [21-25].

Whereas the effects of e.g. information or labelling in nutrition policy have been addressed by a considerable number of studies^ii^, the number of studies addressing the potentials of economic incentive instruments in nutrition policy is relatively limited^iii^. The few existing examples of empirical studies include [40], which analyses VAT-increases for foods containing saturated fats or cholesterol, and [42], which analyses the effects on food consumption and tax revenues of a VAT reduction on fruits and vegetables. These analyses suggest that such VAT-adjustments may have considerable effects on the intake of nutrients. Similar findings are obtained in a Danish study, which also finds different food demand responses in different socio-demographic groups [17].

Despite this number of existing theoretical and empirical studies on food taxation, there seems to be no empirical studies dealing with the design of food taxation/subsidization instruments and thus the potentials for optimizing the efficiency of such instruments. A key result from the economic literature on regulation is that the cost effectiveness of a policy instrument depends on the instrument's precision in targeting the considered problem. The more precisely the regulation targets the problem the smaller will be substitution effects (e.g. substitution from one unhealthy food type to another) etc., which may undermine the effectiveness of the regulation. On the other hand, the cost-effectiveness also depends on the affected agents' possibilities to adjust to the regulation, and thus save costs. This relationship is also valid with regard to regulating the diet in order to improve the future health of the population and the public health care costs: the more precisely the measures can be targeted towards these objectives, the more cost effective are the measures.

One type of economic policy measure with regard to the composition of diet is to impose taxes or subsidies on specific foods, for instance a VAT reduction on selected groups of food, like fruits and vegetables. A policy measure like this will provide consumers with an economic incentive to increase their intake of fruits and vegetables at the cost of other foods like meat, fish and dairy products and thus lead to a less fat-intensive diet. On the other hand, the precision with respect to future health condition and public spending is more uncertain, because the health-enhancing effect varies across fruits and vegetables, and price-induced adjustments in diet composition may include changes, which are not desirable from a nutritional point of view (e.g. decreased consumption of some other healthy foods). Furthermore, potential effects on physical activity are not taken into account.

Another type of economic measure is to impose taxes on specific detrimental components in the food commodities, e.g. the content of saturated fats or sugar like the scheme proposed by Marshall [40]. Compared with the former type of economic measure, such a tax will be more closely connected to a final aim of improved future health. On the other hand, the administration of such a tax may be more costly due to higher requirements for documentation etc.

Other types of economic regulation might be to increase the economic incentives to physical activity, for instance by public support to sports activities, or to impose economic incentives with respect to the consequences of unhealthy lifestyle, for example a tax on the individual's weight or BMI [20], or higher degree of payment on health care costs, which can be traced back to unhealthy lifestyle or overweight, possibly through insurance schemes where the premium depends on lifestyle etc.

The precision of taxes and subsidies on foods may be lower than for other measures, especially if very detailed objectives are pursued, e.g. improving the diet of selected "risk segments" of the population. A potential barrier for the effectiveness of taxes and subsidies might be low response to price changes for targeted consumer segments, due to e.g. imperfect information, habits or lack of time. A range of studies document that the level of information varies considerably across groups and such variations in the informational basis may have implications for consumers' choices [35,36]. Furthermore, economic ability or nutritional needs may vary across groups, and the use of economic instruments may lead to undesired regressive distributional effects, implying that poorer consumers are taxed more heavily than richer consumers [43]. Thus, "horizontal" policy measures that affect e.g. the price conditions equally for all consumers may give rise to undesired distributional effects that could be avoided by using more detailed "selective" regulation targeted at selected groups of consumers.

In addition to the issue of targeting economic incentive instruments, it should be noted that market prices result from the combination of demand and supply relations. Thus, the less price elastic is the supply of a food commodity, the less will be the market price response to a considered food tax change. The fact that Denmark is member of the European Union implies that the food supply in Denmark is subject to international competition, suggesting that food supplies are relatively price elastic. This is supported by transmission studies, where variations in domestic prices for many food commodities to a large extent can be explained by price variations in associated markets, indicating that domestic suppliers are facing competition from imported products [44]. To the extent that supply is not perfectly price elastic, an economic measure (e.g. tax reduction) may not be fully transmitted to the consumer price – some of the impact may be absorbed by increased margins in the food supply chain.

## 4. Methodology

**Figure 1:**
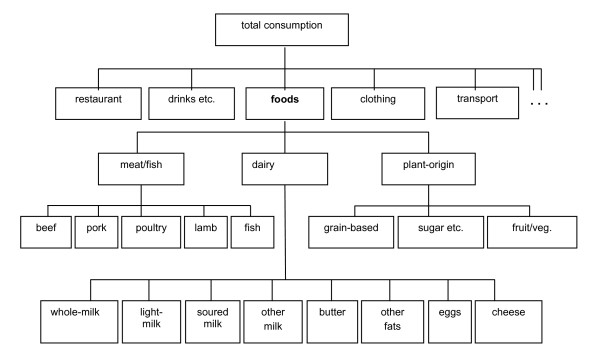
Assumed separability structure in economic model.

In the following, some consequences of using economic regulation are analysed for various economic policy instruments, using Denmark as an illustrative case. The analyses are carried out on the basis of an economic model, which is based on estimated behavioural parameters (reproduced in the appendix), where changes in the consumption of foods are expressed as functions of changes in the relative prices of these foods. Specifically, a dynamic linearized Almost Ideal Demand System [45,21] covering 16 food categories^iv ^and 6 other consumption good commodity groups^v ^was specified and estimated econometrically using aggregate annual data from Statistics Denmark, spanning the period 1972–1996. In order to make econometric estimation feasible, a number of separability assumptions were made and imposed^vi^. The separability structure is shown in figure 1.

For each of the nests, a conditional demand system has been specified and estimated. In the estimation process for each nest, it has been ensured that the estimated parameters conform to standard properties of demand systems (adding-up, linear homogeneity, Slutsky symmetry and concavity). Having estimated all the nested conditional demand systems, it is possible to derive corresponding unconditional demand parameters for all commodities in the system [25,47]. Results of the econometric estimations are presented in the appendix.

Due to the data used for econometric estimation, the estimated parameters represent changes in food consumption measured in fixed-price value terms. In order to assess the nutritional impacts of e.g. changed price relations, there is a need for converting these value estimates into physical quantities. Based on aggregate fixed-price value data and aggregate physical quantity data for the consumption of foods, a matrix for converting value data to physical quantities has been constructed.

From data on physical quantities of individual food components, the intake of various nutrients can be estimated using nutrient coefficients from the Danish food database, which describe the average content of a number of micro- and macro-nutrients in a large range of the most usual food commodities on the Danish market [48]. For the present purpose, these coefficients have been aggregated to the level of detail obtainable in the econometric food demand model. The nutrient coefficients applied in the present study are displayed in table 2.

**Table 2:** Selected aggregate nutrient coefficients

	Fats, total	Saturated fats	Sugar	Fibres
	g/100 g	g/100 g	g/100 g	g/100 g
Milk	1,6	1,11	0,9	0,1
Butter, margarine, other fats	81,4	51,80	0	0,0
Eggs	11,2	3,04	0	0,0
Cheese	16,0	10,40	0	0,0
Meats	11,3	3,04	0	0,0
Fish	1,5	0,34	0	0,0
Grain-based foods	3,7	1,08	0	3,1
Sugar	0,0	0,00	99,9	0,0
Fruit/vegetables/potatoes	0,3	0,16	0	1,7

Source: [48]

The complete model system applied for the analysis is illustrated in [see additional file 1].

## 5. Scenarios and results

The developed model framework is used for analysing various types of economic instruments, which are assumed to give the consumers economic incentives to change their food consumption patterns into more healthy directions. The types of instruments analysed include

• various taxes on nutrients, which are crucial with regard to obesity and nutrition-related diseases: fats, saturated fats [49] and sugar [50]

• various forms of subsidies to nutrients, which are desired to be promoted: fruits and vegetables [51], fibres [52]

• revenue-neutral combinations of taxes and subsidies

7 different regulation scenarios have been specified, cf. table 3. In order to make the scenarios mutually comparable, all scenarios are scaled in a way that their implications for the net economic welfare are equal across the scenarios. The point of departure for this scaling is the welfare loss due to a halved VAT rate for fruits, vegetables and potatoes (from 25% to 12.5%). In the revenue-neutral combination scenarios, the net effect on tax revenue is equal to zero.

**Table 3:** Overview of scenarios

Subsidy scenarios
1: VAT on fruits, vegetables and potatoes halved (from 25 to 12.5%)
2: Subsidy on fibres (approximately 76.40 DKK per kg fibre)
Tax scenarios
3: Tax on all fats (approximately 8.00 DKK per kg. fat)
4: Tax on saturated fats (approximately 14.00 DKK per kg. saturated fat)
5: Tax on sugar (approximately 5.60 DKK per kg sugar)
Revenue neutral combinations of taxes and subsidies
6: Subsidy on fibres and tax on saturated fats and sugar
7: Halved VAT on fruits and vegetables and tax on fats and sugar

The taxes and subsidy rates on nutrients are assumed to affect the consumer food prices according to their content of these specific nutrients [see table 2]. We further assume that food supplies are price elastic, implying that the tax and subsidy changes are fully transmitted to consumer prices. As suggested above, this assumption may be justified by the fact that Denmark is part of the large EU market with several competing food suppliers. On the other hand, a high degree of market concentration in some stages of the food supply chains (e.g. the retail stage), even at the EU level, may imply a less than full impact of tax changes on consumer prices. Hence, whereas the assumption of full transmission of tax changes to consumer prices may lead to some uncertainty regarding the absolute impacts of the different scenarios it is considered less crucial for the comparison between scenarios.

Subsidies based on the fibre content (scenario 2) will benefit most foods of plant origin, as opposed to a tax reduction on fruits and vegetables (scenario 1), which will only benefit consumption of these commodities. A tax on all fats (scenario 3) or saturated fats (scenario 4) will lead to price increases for most foods – and primarily for foods of animal origin. A tax on sugar (scenario 5) will almost exclusively have effects on the prices of sugar, sweets, cakes and fruit yoghurt. To the extent that consumers adjust their consumption patterns to changed price conditions, all the considered scenarios, except scenario 5, are expected to lead to shifts away from animal-origin foods towards plant-origin foods.

The consumer price changes induced by the scenarios are used as input to the economic model in order to determine the effects on the consumption of different food categories and the intake of different nutrients. The calculated effects of the 7 scenarios on food and nutrient intake are shown in table 4.

**Table 4:** Effects on the intake of selected foods and nutritional components, per cent.

Scenario	Subsidy scenarios		Tax scenarios			Combined scenarios	
	1	2	3	4	5	6	7
			
	Fruits/vegetables	Fibres	Total fats	Saturated	Sugar	Nutrients	Commodities
Milk	-1.6	-1.2	-1.6	-1.9	1.2	-1.2	-1.4
Butter and fats	-1.8	-2.5	-12.6	-14.5	2.1	-7.6	-5.1
Cheese	-2.2	-3.0	-7.0	-7.7	2.5	-4.5	-3.3
Eggs	-2.1	-2.9	1.0	3.3	2.5	0.4	-0.5
Meat	0.0	-0.4	-5.4	-3.6	0.2	-1.7	-1.7
Fish	-1.6	-2.5	1.6	1.8	1.9	-0.2	-0.1
Flour, bread etc.	1.3	8.6	1.7	1.9	0.4	7.0	1.7
Sugar	0.2	-3.3	6.4	6.4	-15.8	-6.5	-3.1
Potatoes, fruits and vegetables	7.8	5.1	3.3	3.3	1.0	5.5	7.6

							
Fats	-0.7	-0.5	-6.1	-5.9	1.2	-2.5	-2.3
Saturated fats	-1.1	-1.1	-7.2	-7.4	1.4	-3.6	-2.9
Fibres	4.1	6.7	2.3	2.4	0.7	6.1	4.3

Subsidies to the consumption of fruits and vegetables, e.g. in terms of reduced VAT (scenario 1) will induce an increase in the consumption of these foods, at the cost of a range of other foods, including dairy products, eggs and fish. A subsidy to the content of fibres in the foods (scenario 2) leads to an increase in the consumption of fibre-rich foods: flour/bread, potatoes, fruit and vegetables, mainly at the cost of dairy products, eggs and fats.

At the bottom of table 4, the subsidies' calculated effects on the intake of fats, saturated fats and fibres are shown. Both subsidy scenarios have a reducing effect on the intake of fats and a stimulating effect on the intake of fibres. The results show that the choice of taxation object has implications for these effects. For example, the effect on intake of fibres is significantly higher if a tax reduction targets fibres per se, rather than fruits and vegetables. The effects of a fibre subsidy on the fibre intake are possibly underestimated, because the subsidy induces consumers to substitute low-fibre vegetables towards high-fibre vegetables – an effect that has not been accounted for in the calculations due to the level of aggregation in the econometric model.

A tax on all fats in the foods (scenario 3) leads to a reduction in the consumption of all food categories of animal origin, except eggs. The tax induces a relatively strong reduction in the consumption of fats (butter, margarine, oils etc.) and cheese, and to some extent also the consumption of meats, and these foods are replaced by fish, fruits and vegetables, bread and especially sugar. Thus, although the tax on fats has some desired effects on the consumption of fats, it also has some undesired effects in terms of the consumption of sugar. If a fat tax is only directed towards the foods' contents of saturated fats (scenario 4), the picture changes slightly, compared with scenario 3. The reducing effect on the consumption of fats and cheese (which have a high content of saturated fats) is 10–15 per cent stronger. In contrast to taxes on fats, a tax on sugar (scenario 5) only reduces the consumption of sugar^vii^, but induces increases in the consumption of other food categories, including the intake of fats.

The estimated effects on the consumption of individual types of foods in the tax scenarios (3–5) are presumably over-estimated due to consumers' option of changing towards e.g. more low-fat varieties of the individual foods. By shifting from e.g. high-fat milk products towards more low-fat milk products, the consumer may avoid part of the price increase due to the tax, and may thus be less likely to reduce the overall consumption of milk than the above results suggest. For example, by means of estimated detailed price elasticities, it has been calculated that the considered fat tax will lead to an 8–10 per cent reduction in the average fat content in consumed fluid milk, because consumers replace high-fat milk with more low-fat varieties [53]. On the other hand, the effects on fat intake may be underestimated due to these within-aggregate substitution effects.

Combinations of tax reductions on fibres or fruits and vegetables on the one hand, and increased taxes on the most unhealthy fats on the other hand (scenarios 6 and 7) are seen to have desirable effects on the intake of fruit and vegetables, and thus the amount of fibres, while at the same time reducing the intake of fats and sugar. With regard to objectives of reducing the intake of fats and sugar and increasing the intake of fibres, scenario 6 is up to 40 per cent (and for sugar even more than 100 per cent) more effective than scenario 7.

The scenarios also have economic implications for consumers and the government budget, and hence for society as a whole. A measure of the welfare loss is the sum of lost consumers' surplus and net revenue losses for the government. The loss in consumers' surplus is measured in terms of equivalent variation, which measures the food budget change necessary to obtain the initial utility level at the changed prices, taking into account the changed composition of consumption. For instance, if the price of one commodity increases, there will be a need for a budget increase in order to obtain the same utility level as before the price increase. The estimated consequences of the considered scenarios on consumers' surplus, government revenues and economic net welfare are displayed in table 5.

**Table 5:** Economic consequences of economic food policy instruments

Scenario	Subsidy scenarios		Tax scenarios			Combined scenarios	
	1	2	3	4	5	6	7
			
	Fruits/vegetables	Fibres	Total fats	Saturated	Sugar	Nutrients	Commodities
Million DKK							
Consumers' surplus	1094	1555	-1647	-1461	-1242	-41	-41
Net tax revenue	-1134	-1596	1606	1420	1201	0	0
Net welfare cost	41	41	41	41	41	41	41

**DKK/household**							
Consumers' surplus	482	685	-726	-644	-547	-18	-18
Net welfare cost	18	18	18	18	18	18	18

As mentioned above, the 7 scenarios are scaled to yield the same welfare loss (41 million DKK – corresponding to 5.5 million euros – per year) in order to make the scenarios comparable. However, the distribution of this loss between consumers and the government sector varies considerably across scenarios. Thus, a general fat tax (scenario 3) implies a relatively large redistribution from consumers towards the public sector, whereas the redistribution effect of a sugar tax (scenario 5) is less, because the sugar tax affects a smaller share of the food budget.

As expected, the redistributive effect goes in the opposite direction in the two subsidy scenarios, where consumers gain while the government sector suffers a revenue loss. The extent of redistribution is larger for a fibre subsidy (scenario 2) than for the VAT reduction on fruit and vegetables (scenario 1), as the fibre subsidy concerns a larger share of the food budget than fruits and vegetables. Due to construction of the scenarios, the two revenue-neutral combination scenarios (scenario 6 and 7) only affect the consumers, and the welfare loss equals the loss of consumers' surplus.

As was the case with the consumption responses above, the indicated economic effects of taxes are probably over-estimated, because consumers to some extent are able to reduce tax payments beyond those represented in the price elasticities by shifting toward "light" varieties of the products, e.g. from whole-milk to skimmed milk or from high-fat towards low-fat cheeses [53]. On the other hand, the revenue effect of a fibre subsidy is probably under-estimated, because consumers will tend to substitute towards more fibre-rich (and thus more eligible for subsidies) food varieties, when the prices of these are reduced as a consequence of the subsidy.

Consumers' possibilities for substitution between foods and other consumption goods are ignored in the calculations. To the extent such substitution takes place, the costs are over-estimated. However, this is not considered to have serious implications for the comparisons across regulation scenarios in the present context.

As mentioned, an assessment of the cost-effectiveness of the considered regulations is based on a comparison of the economic consequences in table 5 with the nutrient intake effects in table 4. A difficulty in this respect is however the multidimensional character of the nutritional effects (fats, sugar, fibres, etc.). Which of two tax instruments is the most cost-effective from an overall perspective depends on the weighting of the respective nutritional effects. From table 4 it is however seen that a combined regulation, where the instruments are specifically targeted towards the critical nutritional components (scenario 6), has a relatively strong impact on the intake of all the considered components. So even if a precise evaluation of the relative cost-effectiveness of the considered instruments is difficult, there seems to be no doubt that the cost-effectiveness is relatively high for this combination of economic regulation instruments.

## 6. Discussion and conclusion

This paper has analysed the use of differently targeted incentive-based regulation instruments in nutritional policy by quantitative simulations, where nutritional effects of various economic instruments are compared with the associated welfare costs. The importance of selecting objects for regulation as close to the final goals as possible was underlined in the theoretical discussion and the obtained quantitative results support this statement. Hence, the effectiveness with regard to the considered nutritional variables is 10–30 per cent higher in a scenario, which targets critical nutrients (saturated fats, fibres and sugar), than in a scenario, where the targeting is more indirect, in that the regulation targets the consumption of foods like fats, sugar, fruit and vegetables rather than the intake of underlying nutrients. Moreover, the quantitative results illustrate that if the considered nutritional component is e.g. fibres, the strongest effect at a given cost is obtained by targeting the subsidies directly on the fibre content.

The demonstrated effect on food consumption is only an indirect measure of the long-term health effects of the considered food taxation scenarios. The direct health effects depend on the relationships between diet and lifestyle-related illnesses – relationships that may often be highly complex^viii^. However, to the extent that it is possible to measure the effect of a changed diet for the development of such illnesses it is also possible to address such effects in the cost-effectiveness evaluations.

Above, the effects of different economic measures are compared with each other. It will furthermore be possible and relevant to compare these effects with effects of other types of regulation, for example the obtainable nutritional impacts of information campaigns at an annual welfare cost of 41 million DKK.

The issue of administrative problems and costs has only to a limited extent been dealt with in the present study^ix^. It is evident that the administrative cost will differ between the considered regulation instruments. Taxes or subsidies on underlying nutrients like saturated fats or fibres will be more demanding with respect to documentation and control than e.g. a VAT reduction on fruits and vegetables. Administrative costs are thus expected to be higher in scenario 6 than in scenario 7. There will also be differences as to where in the food supply chain the instruments can be implemented. New taxes or subsidies may further give rise to border trade issues and circumvention in terms of increased farm-gate sales etc.

Econometrically estimated parameters as those applied in the above analysis are subject to uncertainty for two reasons: uncertainty due to applied assumptions and statistical uncertainty. A crucial assumption in the present study is the imposed separability structure, which restricts the substitution patterns between commodities. For example, the estimated substitution/complementarity behaviour between bread and butter is restricted to be similar to the pattern between plant-origin and dairy products in general. It is unclear, whether the separability assumptions lead to over- or underestimation of the cross-price elasticities, but the implications of the assumptions for own-price elasticities are considered to be limited. As the own-price elasticities in general constitute the major share of the effects for most commodities, the quantitative analyses below are considered to yield reasonable orders of magnitude, despite the uncertainties induced by the assumed separability structure. In the appendix, the statistical uncertainty of the estimated price elasticities is assessed in terms of estimated standard deviations of the elasticities.

The above quantitative analyses abstract from supply-side adjustments. To the extent food supplies are not perfectly price elastic (e.g. due to imperfect competition, or joint production), the price impacts of changed food taxes may be overestimated in the above calculations. Furthermore, price responses to tax changes may by asymmetric^x^, i.e tax increases are more likely to be reflected in consumer prices than tax decreases. Such imperfections or asymmetries in price responses may not be easily observable, as they can be disguised in various innovative ways, e.g. "meal deals" in the fast food industry, where costs are bundled together, thereby hiding the costs of individual ingredients. As the stages of many Danish food supply chains are characterised by high degrees of concentration [55], this may be a relevant risk, although Denmark's participation in the European Union – and thus potential competition from imported products – should prevent food supply firms' excessive exploitation of market power, beyond what can be explained by e.g. local preferences, transportation costs etc.

Also adjustments due to changed government revenues have been ignored. If a food tax yields a net revenue, this will in principle enable lowering other taxes and hence some of the distorting effects on e.g. labour supply caused by these taxes.

In principle, the introduction of economic regulation implies the same changes in conditions for all consumers, and thus does not yield the possibility to target the regulation towards segments where the needs for adjustment are the largest. In some cases, economic instruments may only be effective for some of the relevant segments, whereas other segments are only affected to a limited extent. If the aim is to improve the food habits and health conditions or all segments with unhealthy food habits, economic instruments alone may not be sufficient, but they may contribute to an improvement in most consumer segments and thus serve as a supplement to other policy initiatives, e.g. information campaigns or school-meal programmes.

The current quantitative study has not addressed distributional effects of the considered food taxation schemes. According to figures from the Statistics Denmark Consumption Survey, food expenditure constitutes around 16 per cent of total disposable income for low-income households and 7 per cent for high-income households. Hence, the relative impact on real disposable income in low-income households may be around twice the impact in high-income households, although the exact ratio will depend on differences in food consumption patterns and price responsiveness in food consumption. People in lower social classes tend to have more unhealthy diets in terms of high intake of fats and sugar and low intake of fibres, which implies that a tax on e.g. fats or sugar will affect their food expenditure more significantly than it will in higher social classes [18].

These distributional concerns have without doubt been among the dominating reasons for decision makers' apparent reluctance to introduce differentiated food taxes as an instrument to prevent obesity so far. Thus, more knowledge about the impacts of different instruments on such distributional effects is needed. However, also other concerns may have hampered the use of food tax differentiation, including administrative concerns, lack of knowledge about the economic and health consequences of differentiated food taxes (which are considerably more complex and less well-documented than for e.g. tobacco) as well as political concerns. Hence, although studies like the present one hopefully sheds some light on the potentials of an appropriate design of food tax instruments, there are still a number of scientific as well as political challenges, that have to be met before the introduction of differentiated food taxes for dietary regulation is likely.

If the use of economic instruments in nutrition policy is to be increased, the possible interactions between such instruments and other policies, including price support measures as those in the European Common Agricultural Policy, food safety policy etc. affecting food price formation and demand, should also be taken into consideration. Whether possible distortions caused by these policies are amplified or neutralised as a consequence of nutrition-related taxes or subsidies depends on the specific range of instruments applied, and this issue may an object for further research.

## Competing interests

The author(s) declare that they have no competing interests.

## Appendix. Econometrically estimated food demand parameters

### Stage 1. Aggregate demand system

At the most aggregate level, an AID demand system comprising foods, beverages and tobacco, eating at restaurants, clothing, dwelling, transports and other consumables is estimated. For each of these commodity aggregates, an equation expressing the commodity's share of the total consumption budget as a linear function of the natural logarithms of the respective prices, *p*, the natural logarithm of the aggregate real consumption budget, *m/P*,, and lagged budget shares is estimated. The variable *m *represents the nominal consumption budget, whereas the variable *P *represents an aggregate of the underlying commodity prices. Results of this estimation (with standard deviations in parentheses) are given in the expression.

wfood,t=2.0975(0.5877)+0.0641(0.0806)⋅ln⁡(pfood,t)+0.0184(0.0184)⋅ln⁡(pbeverages,t)−0.0048(0.0088)⋅ln⁡(prestaurant,t)+0.0693(0.0391)⋅ln⁡(pclothing,t)+0.0373(0.0131)⋅ln⁡(pdwelling,t)−0.0296(0.0438)⋅ln⁡(ptransport,t)+0.0100(0.0070)⋅ln⁡(pother,t)−0.1718(0.0529)⋅ln⁡(mconsumption,t/Pconsumption,t)−0.1920(0.2349)wfood,t−1−0.3596(0.2075)⋅wfood,t−2σ^=0.0029,R2=0.978,DW=2.122
 MathType@MTEF@5@5@+=feaafiart1ev1aaatCvAUfKttLearuWrP9MDH5MBPbIqV92AaeXatLxBI9gBaebbnrfifHhDYfgasaacH8akY=wiFfYdH8Gipec8Eeeu0xXdbba9frFj0=OqFfea0dXdd9vqai=hGuQ8kuc9pgc9s8qqaq=dirpe0xb9q8qiLsFr0=vr0=vr0dc8meaabaqaciaacaGaaeqabaqabeGadaaakqaabeqaauaabeqaceaaaeaacqWG3bWDdaWgaaWcbaGaemOzayMaem4Ba8Maem4Ba8MaemizaqMaeiilaWIaemiDaqhabeaaaOqaaaaafaqabeGabaaabaGaeyypa0dabaaaauaabeqaceaaaeaacqaIYaGmcqGGUaGlcqaIWaamcqaI5aqocqaI3aWncqaI1aqnaeaacqGGOaakcqaIWaamcqGGUaGlcqaI1aqncqaI4aaocqaI3aWncqaI3aWncqGGPaqkaaqbaeqabiqaaaqaaiabgUcaRaqaaaaafaqabeGabaaabaGaeGimaaJaeiOla4IaeGimaaJaeGOnayJaeGinaqJaeGymaedabaGaeiikaGIaeGimaaJaeiOla4IaeGimaaJaeGioaGJaeGimaaJaeGOnayJaeiykaKcaauaabeqaceaaaeaacqGHflY1cyGGSbaBcqGGUbGBcqGGOaakcqWGWbaCdaWgaaWcbaGaemOzayMaem4Ba8Maem4Ba8MaemizaqMaeiilaWIaemiDaqhabeaakiabcMcaPaqaaaaafaqabeGabaaabaGaey4kaSIaeGimaaJaeiOla4IaeGimaaJaeGymaeJaeGioaGJaeGinaqdabaGaeiikaGIaeGimaaJaeiOla4IaeGimaaJaeGymaeJaeGioaGJaeGinaqJaeiykaKcaauaabeqaceaaaeaacqGHflY1cyGGSbaBcqGGUbGBcqGGOaakcqWGWbaCdaWgaaWcbaGaemOyaiMaemyzauMaemODayNaemyzauMaemOCaiNaemyyaeMaem4zaCMaemyzauMaem4CamNaeiilaWIaemiDaqhabeaakiabcMcaPaqaaaaafaqabeGabaaabaGaeyOeI0IaeGimaaJaeiOla4IaeGimaaJaeGimaaJaeGinaqJaeGioaGdabaGaeiikaGIaeGimaaJaeiOla4IaeGimaaJaeGimaaJaeGioaGJaeGioaGJaeiykaKcaauaabeqaceaaaeaacqGHflY1cyGGSbaBcqGGUbGBcqGGOaakcqWGWbaCdaWgaaWcbaGaemOCaiNaemyzauMaem4CamNaemiDaqNaemyyaeMaemyDauNaemOCaiNaemyyaeMaemOBa4MaemiDaqNaeiilaWIaemiDaqhabeaakiabcMcaPaqaaaaaaeaafaqabeGabaaabaGaey4kaSIaeGimaaJaeiOla4IaeGimaaJaeGOnayJaeGyoaKJaeG4mamdabaGaeiikaGIaeGimaaJaeiOla4IaeGimaaJaeG4mamJaeGyoaKJaeGymaeJaeiykaKcaauaabeqaceaaaeaacqGHflY1cyGGSbaBcqGGUbGBcqGGOaakcqWGWbaCdaWgaaWcbaGaem4yamMaemiBaWMaem4Ba8MaemiDaqNaemiAaGMaemyAaKMaemOBa4Maem4zaCMaeiilaWIaemiDaqhabeaakiabcMcaPaqaaaaafaqabeGabaaabaGaey4kaSIaeGimaaJaeiOla4IaeGimaaJaeG4mamJaeG4naCJaeG4mamdabaGaeiikaGIaeGimaaJaeiOla4IaeGimaaJaeGymaeJaeG4mamJaeGymaeJaeiykaKcaauaabeqaceaaaeaacqGHflY1cyGGSbaBcqGGUbGBcqGGOaakcqWGWbaCdaWgaaWcbaGaemizaqMaem4DaCNaemyzauMaemiBaWMaemiBaWMaemyAaKMaemOBa4Maem4zaCMaeiilaWIaemiDaqhabeaakiabcMcaPaqaaaaafaqabeGabaaabaGaeyOeI0IaeGimaaJaeiOla4IaeGimaaJaeGOmaiJaeGyoaKJaeGOnaydabaGaeiikaGIaeGimaaJaeiOla4IaeGimaaJaeGinaqJaeG4mamJaeGioaGJaeiykaKcaauaabeqaceaaaeaacqGHflY1cyGGSbaBcqGGUbGBcqGGOaakcqWGWbaCdaWgaaWcbaGaemiDaqNaemOCaiNaemyyaeMaemOBa4Maem4CamNaemiCaaNaem4Ba8MaemOCaiNaemiDaqNaeiilaWIaemiDaqhabeaakiabcMcaPaqaaaaafaqabeGabaaabaGaey4kaSIaeGimaaJaeiOla4IaeGimaaJaeGymaeJaeGimaaJaeGimaadabaGaeiikaGIaeGimaaJaeiOla4IaeGimaaJaeGimaaJaeG4naCJaeGimaaJaeiykaKcaauaabeqaceaaaeaacqGHflY1cyGGSbaBcqGGUbGBcqGGOaakcqWGWbaCdaWgaaWcbaGaem4Ba8MaemiDaqNaemiAaGMaemyzauMaemOCaiNaeiilaWIaemiDaqhabeaakiabcMcaPaqaaaaaaeaafaqabeGabaaabaGaeyOeI0IaeGimaaJaeiOla4IaeGymaeJaeG4naCJaeGymaeJaeGioaGdabaGaeiikaGIaeGimaaJaeiOla4IaeGimaaJaeGynauJaeGOmaiJaeGyoaKJaeiykaKcaauaabeqaceaaaeaacqGHflY1cyGGSbaBcqGGUbGBcqGGOaakcqWGTbqBdaWgaaWcbaGaem4yamMaem4Ba8MaemOBa4Maem4CamNaemyDauNaemyBa0MaemiCaaNaemiDaqNaemyAaKMaem4Ba8MaemOBa4MaeiilaWIaemiDaqhabeaakiabc+caViabdcfaqnaaBaaaleaacqWGJbWycqWGVbWBcqWGUbGBcqWGZbWCcqWG1bqDcqWGTbqBcqWGWbaCcqWG0baDcqWGPbqAcqWGVbWBcqWGUbGBcqGGSaalcqWG0baDaeqaaOGaeiykaKcabaaaauaabeqaceaaaeaacqGHsislcqaIWaamcqGGUaGlcqaIXaqmcqaI5aqocqaIYaGmcqaIWaamaeaacqGGOaakcqaIWaamcqGGUaGlcqaIYaGmcqaIZaWmcqaI0aancqaI5aqocqGGPaqkaaqbaeqabiqaaaqaaiabdEha3naaBaaaleaacqWGMbGzcqWGVbWBcqWGVbWBcqWGKbazcqGGSaalcqWG0baDcqGHsislcqaIXaqmaeqaaaGcbaaaauaabeqaceaaaeaacqGHsislcqaIWaamcqGGUaGlcqaIZaWmcqaI1aqncqaI5aqocqaI2aGnaeaacqGGOaakcqaIWaamcqGGUaGlcqaIYaGmcqaIWaamcqaI3aWncqaI1aqncqGGPaqkaaqbaeqabiqaaaqaaiabgwSixlabdEha3naaBaaaleaacqWGMbGzcqWGVbWBcqWGVbWBcqWGKbazcqGGSaalcqWG0baDcqGHsislcqaIYaGmaeqaaaGcbaaaaaqaaiqbeo8aZzaajaGaeyypa0JaeGimaaJaeiOla4IaeGimaaJaeGimaaJaeGOmaiJaeGyoaKJaeiilaWIaemOuai1aaWbaaSqabeaacqaIYaGmaaGccqGH9aqpcqaIWaamcqGGUaGlcqaI5aqocqaI3aWncqaI4aaocqGGSaalcqWGebarcqWGxbWvcqGH9aqpcqaIYaGmcqGGUaGlcqaIXaqmcqaIYaGmcqaIYaGmaaaa@C5B4@

### Stage 2. Allocation of the aggregate food consumption budget

The budget for food consumption is allocated into three food categories: dairy products (including fats and eggs), meat and fish, and plant-origin foods (fruits, vegetables, sugar, grain-based products etc.). Due to adding-up, the third equation is dropped from the estimation (but its parameters can be derived from the other two equations).

### Stage 3. Allocation of food categories into food sub-categories

The third stage of the assumed budget allocation process consists of three sub-processes – one for each of the food categories in stage 2. Hence, the budget for dairy commodities is allocated to 8 commodities: wholemilk, light/skimmed milk, sour milk/yoghurt, other fluid milk, butter, margarine, eggs and cheese, as a function of the respective logarithmic prices of these foods, the total dairy consumption budget and lagged budget shares representing rigidities in the consumption patterns (habits etc.) The budget for meat and fish is allocated to 5 commodities: beef, pork, poultry meat, lamb and fish, and the consumption budget for plant-origin foods is allocated to 3 commodity groups: grain-based foods (flour, bread, cereals, pasta, rice), sugar and fruits and vegetables. Unfortunately, the level of detail in these commodities could not be higher due to data limitations.

Estimation statistics for the estimated models are presented in [see additional file 2].

The estimated parameters have been combined with average budget shares in order to calculate a matrix of uncompensated price elasticities for food demand, distributed on 16 food categories. The resulting matrix of uncompensated price elasticities is displayed in [see additional file 3] (Asymptotic standard deviations in small fonts).

The price elasticity estimates are more or less in line with other estimates from the literature. For example, a study finds price elasticities for fruits and vegetables in the range -0.6 to -0.9 for Norway [24], whereas another study estimates the price elasticity for fruits and vegetables to be -0.77 for Denmark [22]. Price elasticity estimates concerning dairy products for Norway [25] and for Denmark [22] and concerning meats from Norway [23], U.S. [56], U.K. [57] and Belgium [58] are also in line with the estimates applied in the present study. It should however be mentioned, that studies finding somewhat lower price elasticity estimates also exist, e.g. [21].

## Note

^i ^Many people previously were paid to be physically active at work because many tasks had a physical content.

^ii ^For examples, see [8,26-36]

^iii ^The few existing examples include [8,17,37-42].

^iv ^Whole-milk, light milk, soured milk, other milk, butter, other fats, eggs, cheese, beef, pork, poultrymeat, lamb, fish, flour/bread, sugar and fruits/vegetables/potatoes

^v ^Drinks/tobacco, restaurant, clothing, housing, transportation and other goods

^vi ^The separability structure has been specified based on general intuition, as there have been too few observations to perform statistical tests to identify the most appropriate structure, following approaches like [46]. Even if the number of observations were sufficient for performing statistical separability tests, it is however expected to be difficult to identify a clear separability structure on the considered level of aggregation. Although the selected structure may seem appealing, it should be mentioned that the structure is not beyond discussion, as there may be specific substitutions or complementarities between individual commodities within the groups, e.g. bread and butter, breakfast cereals and milk. Prepared and processed meals pose another challenge to the separability assumption, as such meals may include commodities from different nests. In the model, processed foods are categorized according to the international COICOP classification system, which implies that e.g. pizzas and sandwiches are included in the group of grain-based foods. This in turn implies that a lower price on e.g. vegetables may also imply lower average prices on grain-based products, because sandwiches become cheaper. In Denmark, such meals however still comprise a relatively low share of total food consumption.

^vii ^It should be noted that the estimated effects of a sugar tax is relatively imprecise, as the consumption of soft drinks is not modelled in detail.

^viii ^For example, obesity increases the risk of diabetes with 5–10 times and doubles the risk of cardiovascular diseases compared to normal weight [15].

^ix ^A thorough discussion of such aspects is given in [41].

^x ^See e.g. [54] for a review of litterature on asymmetric price responses.

## Supplementary Material

Additional file 1 Integrated model system for quantitative analysis. Structure of the integrated demand-nutrient model with interaction between consumers' economic behaviour and nutritional consequences. Click here for file

Additional file 2 Econometric estimation statistics. Key indicators of statistical performance of econometric model for analysing food demand behaviour. Click here for file

Additional file 3 Estimated food demand elasticities. Estimated uncompensated price and income elasticities for Danish demand of major food categories. Click here for file
