# Supplementary material for: Cost-effective design of economic instruments in nutrition policy
Source: Int J Behav Nutr Phys Act. 2007 Apr 4;4:10. doi: 10.1186/1479-5868-4-10 (PMC1855063; doi:10.1186/1479-5868-4-10)
Supplement: Additional file 1 — Integrated model system for quantitative analysis. Structure of the integrated demand-nutrient model with interaction between consumers' economic behaviour and nutritional consequences. [file 1479-5868-4-10-S1.doc]

The consequences of economic instruments are analysed by means of an economic model containing the following three components:

- quantitative description of the interrelations between quantities of final food products and quantities of ingredients (input-output coefficients)

- quantitative description of the contents of various nutrients in the ingredients in terms of nutrient coefficients

- quantitative description of the demand for final food products, including the price responsiveness of this demand (price elasticities).

Tax/subsidy on nutrient

Prices of ingredients

Price on final food products

coefficients for ingredients’ content of nutrients

input-output

coefficients

price

elasticities

Consumption of final food products

Use of ingredients

Nutrient

intake

A tax on a specific nutrient affects the prices of the ingredients containing the nutrient. Provided the price changes on ingredients, the change in the prices of final food products can be determined using input-output coefficients, which describe the correspondence between 1 kg final food product (e.g. bread) and the quantities of different ingredients (e.g. flour, fats, milk etc.). The effects of changed prices on final food products on the demand for foods is calculated using econometrically estimated price elasticities. Changes in the consumption of final food products can be converted to changes in the consumption of ingredients using the above-mentioned input-output coefficients. Together with coefficients for the nutrient contents in ingredients, the consumers’ intake of nutrients can be determined.
