# Supplementary material for: Cost-effective design of economic instruments in nutrition policy
Source: Int J Behav Nutr Phys Act. 2007 Apr 4;4:10. doi: 10.1186/1479-5868-4-10 (PMC1855063; doi:10.1186/1479-5868-4-10)
Supplement: Additional file 2 — Econometric estimation statistics. Key indicators of statistical performance of econometric model for analysing food demand behaviour. [file 1479-5868-4-10-S2.doc]

|  | MSE | R2 | Durbin Watson |
| --- | --- | --- | --- |
| **Overall food demand system** |  |  |  |
| Dairy budget share | 0.0044 | 0.8621 | 1.9887 |
| Meat/fish budget share | 0.0074 | 0.8175 | 2.0735 |
| **Dairy demand system** |  |  |  |
| Whole milk | 0.0051 | 0.9937 | 2.0554 |
| Lean milk | 0.0055 | 0.9939 | 1.7949 |
| Sour milk | 0.0024 | 0.9748 | 1.9908 |
| Other milk | 0.0052 | 0.9146 | 1.7009 |
| Butter | 0.0072 | 0.9350 | 1.5648 |
| Other fats | 0.0050 | 0.9758 | 2.1385 |
| Eggs | 0.0031 | 0.9750 | 2.7864 |
| **Meat/fish demand system** |  |  |  |
| Beef | 0.0089 | 0.9010 | 1.9721 |
| Pork | 0.0105 | 0.9407 | 1.5283 |
| Poultry meat | 0.0073 | 0.9502 | 1.9277 |
| Lamb | 0.0065 | 0.6520 | 1.3075 |
| **Crop products demand system** |  |  |  |
| Flour, bread etc. | 0.0068 | 0.8674 | 2.0208 |
| Sugar | 0.0062 | 0.7104 | 1.9838 |
