# Supplementary material for: Cost-effective design of economic instruments in nutrition policy
Source: Int J Behav Nutr Phys Act. 2007 Apr 4;4:10. doi: 10.1186/1479-5868-4-10 (PMC1855063; doi:10.1186/1479-5868-4-10)
Supplement: Additional file 3 — Estimated food demand elasticities. Estimated uncompensated price and income elasticities for Danish demand of major food categories. [file 1479-5868-4-10-S3.doc]

|  | Elasticity with respect to price of: | | | | | | | | | | | | | | | | Budget |
| --- | --- | --- | --- | --- | --- | --- | --- | --- | --- | --- | --- | --- | --- | --- | --- | --- | --- |
|  | Whole milk | Lean milk | Sour milk | Other milk | Butter | Other fats | Eggs | Cheese | Beef | Pork | Poultry meat | Lamb | Fish | Flour, bread etc. | Sugar | Fruits, vegetables |  |
| Whole milk | -1,125 | 0,233 | 0,133 | -0,184 | 0,267 | -0,430 | 0,180 | 0,035 | 0,083 | 0,207 | 0,043 | 0,003 | 0,058 | 0,227 | 0,202 | 0,206 | 0,222 |
|  | 0,230 | 0,191 | 0,093 | 0,083 | 0,159 | 0,092 | 0,109 | 0,358 | 0,128 | 0,314 | 0,068 | 0,010 | 0,089 | 0,297 | 0,264 | 0,270 | 0,043 |
| Lean milk | 0,108 | -0,439 | -0,410 | -0,263 | 0,122 | -0,073 | 0,026 | -0,233 | 0,079 | 0,196 | 0,041 | 0,002 | 0,055 | 0,217 | 0,193 | 0,197 | 0,201 |
|  | 0,182 | 0,199 | 0,086 | 0,104 | 0,166 | 0,113 | 0,110 | 0,315 | 0,112 | 0,275 | 0,060 | 0,009 | 0,078 | 0,264 | 0,234 | 0,240 | 0,039 |
| Sour milk products | 0,342 | -0,604 | -0,739 | -0,186 | -0,405 | 0,167 | -0,340 | 1,232 | 0,083 | 0,206 | 0,043 | 0,003 | 0,058 | 0,226 | 0,201 | 0,205 | 0,220 |
|  | 0,182 | 0,185 | 0,154 | 0,151 | 0,176 | 0,153 | 0,157 | 0,258 | 0,119 | 0,290 | 0,063 | 0,010 | 0,082 | 0,283 | 0,251 | 0,257 | 0,040 |
| Other milk products | -0,159 | -0,241 | -0,176 | -0,424 | 0,244 | 0,008 | -0,363 | 0,461 | 0,088 | 0,218 | 0,046 | 0,003 | 0,061 | 0,237 | 0,210 | 0,215 | 0,241 |
|  | 0,109 | 0,123 | 0,080 | 0,095 | 0,120 | 0,091 | 0,092 | 0,198 | 0,142 | 0,348 | 0,075 | 0,011 | 0,098 | 0,326 | 0,290 | 0,297 | 0,046 |
| Butter | 0,212 | 0,211 | -0,310 | 0,070 | -0,767 | -0,294 | 0,117 | -0,265 | 0,090 | 0,224 | 0,047 | 0,003 | 0,062 | 0,242 | 0,215 | 0,220 | 0,251 |
|  | 0,149 | 0,148 | 0,081 | 0,088 | 0,163 | 0,092 | 0,095 | 0,278 | 0,143 | 0,351 | 0,076 | 0,011 | 0,099 | 0,333 | 0,296 | 0,303 | 0,045 |
| Other fats | -0,579 | -0,085 | 0,065 | -0,055 | -0,450 | -0,054 | -0,052 | 0,514 | 0,057 | 0,142 | 0,030 | 0,002 | 0,039 | 0,168 | 0,149 | 0,153 | 0,104 |
|  | 0,210 | 0,218 | 0,120 | 0,178 | 0,249 | 0,187 | 0,141 | 0,318 | 0,093 | 0,230 | 0,049 | 0,005 | 0,064 | 0,190 | 0,169 | 0,173 | 0,037 |
| Eggs | 0,254 | 0,180 | -0,299 | -0,359 | 0,230 | -0,012 | -0,618 | -0,111 | 0,080 | 0,200 | 0,042 | 0,003 | 0,056 | 0,220 | 0,196 | 0,200 | 0,208 |
|  | 0,129 | 0,140 | 0,073 | 0,081 | 0,122 | 0,086 | 0,096 | 0,228 | 0,112 | 0,274 | 0,060 | 0,009 | 0,078 | 0,268 | 0,238 | 0,244 | 0,038 |
| Cheese | -0,167 | -0,270 | 0,153 | -0,064 | -0,350 | 0,120 | -0,234 | -1,007 | 0,084 | 0,208 | 0,043 | 0,003 | 0,058 | 0,227 | 0,202 | 0,206 | 0,222 |
|  | 0,274 | 0,250 | 0,113 | 0,093 | 0,189 | 0,105 | 0,136 | 0,442 | 0,119 | 0,291 | 0,063 | 0,010 | 0,082 | 0,285 | 0,253 | 0,259 | 0,039 |
| Beef | -0,001 | -0,001 | 0,000 | 0,000 | -0,001 | 0,000 | 0,000 | -0,001 | -0,362 | 0,601 | -0,177 | -0,489 | -0,242 | 0,151 | 0,135 | 0,138 | 0,220 |
|  | 0,048 | 0,062 | 0,029 | 0,037 | 0,056 | 0,038 | 0,039 | 0,095 | 0,351 | 0,429 | 0,217 | 0,460 | 0,341 | 0,209 | 0,186 | 0,190 | 0,044 |
| Pork | -0,001 | -0,001 | 0,000 | -0,001 | -0,001 | -0,001 | -0,001 | -0,001 | -0,195 | -1,097 | -0,452 | 0,121 | -0,355 | 0,150 | 0,134 | 0,137 | 0,218 |
|  | 0,047 | 0,061 | 0,029 | 0,036 | 0,055 | 0,037 | 0,039 | 0,093 | 0,744 | 0,821 | 0,511 | 1,205 | 0,580 | 0,205 | 0,182 | 0,187 | 0,043 |
| Poultry meat | 0,001 | 0,001 | 0,001 | 0,001 | 0,001 | 0,001 | 0,001 | 0,002 | -0,004 | 0,470 | -0,520 | 0,429 | -0,602 | 0,162 | 0,144 | 0,147 | 0,242 |
|  | 0,053 | 0,069 | 0,032 | 0,040 | 0,062 | 0,042 | 0,044 | 0,105 | 0,169 | 0,319 | 0,123 | 0,139 | 0,243 | 0,231 | 0,205 | 0,210 | 0,047 |
| Lamb | -0,017 | -0,021 | -0,010 | -0,012 | -0,019 | -0,013 | -0,014 | -0,033 | -15,500 | 10,824 | 7,166 | -1,630 | -0,881 | 0,044 | 0,039 | 0,040 | 0,007 |
|  | 0,365 | 0,471 | 0,223 | 0,270 | 0,420 | 0,281 | 0,303 | 0,732 | 28,508 | 28,513 | 28,486 | 28,505 | 28,575 | 1,252 | 1,112 | 1,137 | 0,433 |
| Fish | 0,001 | 0,001 | 0,001 | 0,001 | 0,001 | 0,001 | 0,001 | 0,002 | -0,163 | 0,616 | -0,523 | -0,042 | -0,270 | 0,161 | 0,143 | 0,146 | 0,239 |
|  | 0,052 | 0,067 | 0,032 | 0,040 | 0,061 | 0,041 | 0,043 | 0,104 | 0,212 | 0,338 | 0,115 | 0,132 | 0,276 | 0,227 | 0,201 | 0,207 | 0,046 |
| Flour, bread etc. | -0,018 | -0,023 | -0,011 | -0,013 | -0,020 | -0,014 | -0,015 | -0,036 | 0,001 | 0,002 | 0,000 | 0,000 | 0,000 | -0,821 | 0,137 | -0,071 | 0,181 |
|  | 0,071 | 0,092 | 0,043 | 0,053 | 0,082 | 0,055 | 0,059 | 0,142 | 0,263 | 0,653 | 0,137 | 0,010 | 0,182 | 0,604 | 0,543 | 0,507 | 0,038 |
| Sugar | -0,019 | -0,024 | -0,011 | -0,014 | -0,022 | -0,014 | -0,016 | -0,038 | -0,002 | -0,005 | -0,001 | 0,000 | -0,002 | 0,205 | -0,922 | 0,060 | 0,168 |
|  | 0,066 | 0,086 | 0,041 | 0,050 | 0,077 | 0,052 | 0,055 | 0,133 | 0,245 | 0,609 | 0,128 | 0,010 | 0,170 | 0,879 | 0,844 | 0,676 | 0,037 |
| Fruits, vegetables | -0,018 | -0,023 | -0,011 | -0,013 | -0,021 | -0,014 | -0,015 | -0,036 | 0,000 | 0,000 | 0,000 | 0,000 | 0,000 | -0,016 | 0,067 | -0,713 | 0,177 |
|  | 0,070 | 0,091 | 0,043 | 0,053 | 0,081 | 0,055 | 0,058 | 0,141 | 0,259 | 0,643 | 0,135 | 0,010 | 0,179 | 0,512 | 0,461 | 0,465 | 0,038 |
